# The association between antibiotics in the first year of life and child growth trajectory

**DOI:** 10.1186/s12887-018-1363-9

**Published:** 2019-01-16

**Authors:** Elizabeth E. Dawson-Hahn, Kyung E. Rhee

**Affiliations:** 1 Department of Pediatrics, University of Washington, Seattle, WA USA; 2 Seattle Children’s Research Institute, Center for Child Health, Behavior and Development, M/S CW8-6, PO Box 5371, Seattle, WA 98145 USA; 3 Department of Pediatrics, UCSD School of Medicine, University of California San Diego, 9500 Gilman Drive, MC 0874, La Jolla, CA, San Diego, CA 92093 USA

**Keywords:** Antibiotics, Pediatrics, Growth, Weight status, Breastfeeding

## Abstract

**Background:**

Antibiotics are frequently prescribed to children, and may be an environmental influence that contributes to the increasing prevalence of childhood obesity. The aim of this study was to examine the effect of antibiotic use in the first year of life on child growth trajectories from birth to age 6 years including significant covariates.

**Methods:**

Data from 586 children in the Infant Feeding Practices II (IFPS II) and 6 year follow-up study (6YFU) were included. Antibiotic exposures, weight and height measurements were collected from birth through the first 12 months, and then again at 6 years. Linear mixed effects growth modeling, controlling for exclusive breastfeeding, socio-demographic factors, smoking during pregnancy, gestational diabetes, and maternal pre-pregnancy weight status, was used to examine the association between antibiotic exposure and child growth trajectories through age 6 years.

**Results:**

The majority of infants (60.58%) did not receive any antibiotics; 33.79% received 1–2 courses and 5.63% received 3 or more antibiotic courses during the first year. In the unadjusted model, children with 1–2 antibiotic exposures had a 0.17 (SE 0.08) higher rate of change in BMI z-score (BMIz) than children without any antibiotics, and children with ≥3 exposures had a 0.42 (SE 0.16) higher rate of change in BMIz (*p* = 0.009). Growth trajectory over time for those who had ≥3 antibiotics was greater than those without any antibiotics (*p* = 0.002).

**Conclusions:**

Efforts to guide the judicious use of antibiotics should continue, particularly in the first year of life.

## Background

Children receive antibiotics in over 20% of ambulatory visits in the United States. [1] Despite the clear benefits of antibiotics for specific illnesses, they can also have unintended consequences including antimicrobial resistance [2] and the development of atopy and inflammatory bowel disease (IBD). [3, 4] Recently, there has been growing interest in the association between antibiotics and increased weight status. [5–13] The association between antibiotic exposure and the development of these chronic diseases is postulated to be through alterations to the gut microbiota. At this time, studies have shown that the make-up of the gut microbiota varies between overweight and normal weight individuals, [14] and that changes in weight are associated with changes in the gut microbiota. [15] Often, a higher proportion of microbiota from the Bacteroidetes phylum are present in lean individuals and a higher proportion of Firmicutes in obese individuals. [16–18] Administration of antibiotics can acutely alter the composition of the gut microbiota, leading to decreased microbial load and phylogenetic diversity, and ultimately the overgrowth of bacteria that have increased capacity to harvest energy from one’s dietary intake and promote excess weight gain. [19–21] In animal studies repeated early antibiotic exposures lead to perturbations in the gut microbiota and sustained changes in the metabolic profiles of mice. [22] These changes can remain for several years and long-term use of antibiotics may lead to permanent changes in the gut microbiota. [23]

Several human studies have suggested an association between childhood antibiotic exposure and weight status. [5–10, 13, 24–26] These studies have occurred in the UK, Finland, Denmark, the Netherlands, Canada, and the US, and have primarily focused on oral antibiotic exposure at < 2 years old and risk of obesity between 2 and 12 years old. While these cohorts have enhanced our understanding of the relationship between antibiotics and child weight status, several of them did not control for key factors known to affect child weight status and growth, [5, 6, 10, 12, 13] such as maternal BMI, gestational diabetes, and breastfeeding status. [27–31] Breastfeeding is associated with the development of a gut microbial pattern that may influence weight gain trajectories and metabolic profiles. Infants who are breastfed have a lower risk of being overweight. Additionally, breastfeeding is associated with both weight status and antibiotic receipt. [32–34] Higher maternal weight status and gestational diabetes are also associated with increased weight status [29–31] and has not been included in many of the previously mentioned studies. [5–7, 10, 12, 13] As such, it is important to control for these variables as we examine the relationship between antibiotic use and early weight gain.

Our goal was to further examine the relationship between antibiotic use in the first 12 months and its effect on growth trajectories during the first 6 years. Using the Infant Feeding Practices II (IFPSII) survey and 6-Year Follow-Up study (6YFU), we aimed to examine growth trajectories using mixed effects linear regression models controlling for many of the covariates associated with childhood obesity and antibiotic use, including breastfeeding and maternal BMI that have not consistently been included in previous analyses.

## Methods

### Study sample

**Fig. 1:**
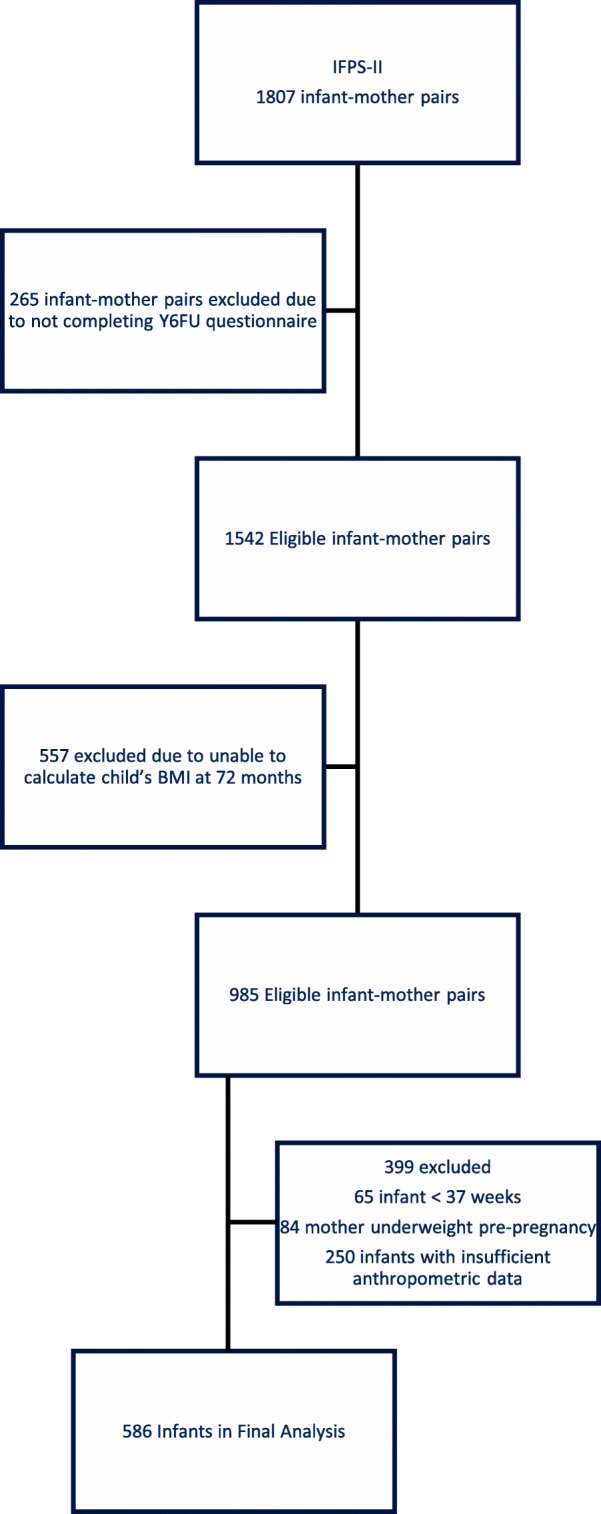
Study cohort diagram

Data from the Infant Feeding Practices Study II (IFPS) [35], which followed mother-infant pairs from late pregnancy through the infants’ first year of life, and the 6 year follow-up study (Y6FU) [36] were included. The study was conducted by the US Food and Drug Administration in collaboration with the Centers for Disease Control and Prevention (CDC) drawing from a nationally distributed consumer opinion panel from May 2005 to June 2007. The study was designed to better understand infant feeding practices and factors that influence infant feeding, infant health, and maternal health and diet. Details regarding the IFPS II have been previously published. [35] Mothers were eligible to participate in the study if they were ≥ 18 years old at the prenatal questionnaire, were having a singleton infant born at ≥35 weeks gestation and if the infant weighed ≥5 pounds at birth. Neither the mothers nor the infants were eligible if they had a medical condition that could impact feeding. Mothers received questionnaires by mail at 7 months gestation, birth, the neonatal time point (~ 3 weeks old), and post-natal months 2, 3, 4, 5, 6, 7, 9, 10, and 12. Initially 1807 women completed questionnaires through the first 12 months of life. In 2012, the 6YFU questionnaire was sent to infant-mother pairs who participated in the IFPS II study, and a total of 1542 questionnaires were completed (Fig. 1). [36]

Of the 1542 mothers who completed the 6YFU questionnaire, only 985 provided the weight, height, and date of their child’s primary care visit in order for 72 month BMI z-score to be calculated. Additional subjects were excluded if infants were less than 37 weeks gestation (*n* = 65) or mothers were underweight (BMI < 18.5) at the pre-pregnancy time point (*n* = 84). Infants who did not have anthropometric data (weight and/or length/height) or the date of the primary care physician visit at 0, 2, 4, 6, 12, and 72 months) were dropped from the analysis. Children with a weight-for-length (WLz) z-score or BMI z-score less than − 5.0 or greater than 5.0 were considered outliers and also dropped from the analysis. As a result, 586 infants were included in the final analysis (Fig. 1). This study was deemed exempt by the Institutional Review Board of the University of California, San Diego.

### Measures

#### Exposure

The use of antibiotics during the first year of life was asked on all questionnaires between 2 months and 12 months of age. Mothers were asked a yes/no question: “Did your baby receive any of the following medicines in the past 2 weeks?” with “antibiotics” being one of the options. [37] Infants had anywhere from 0 to 8 courses of antibiotics in the first year of life. Antibiotic use was then categorized as none, 1–2 courses, and 3 or more courses for the analysis.

#### Outcome

The primary outcome variables were infant WLz at 2, 4, 6 and 12 months and BMI z-score (BMIz) at 72 months. Mothers reported the child’s weight in pounds and length in inches from the child’s 2, 4, 6, 12 and 72 well-child visit to the primary care provider. Overweight/Obese status in children at age 6 years was defined by having a BMI z-score ≥ 1.64 (BMI ≥ 95th percentile per CDC guidelines). [38] The date of each measurement was reported and the child’s age was determined by calculating the difference between the infant’s birth date and the reported date of the primary care visit. Average age at these visits were: 2 months, 68.32 ± 13.85 days; 4 months, 126.10 ± 15.47 days; 6 months, 191.82 ± 19.07 days; 12 months, 362.38 ± 24.28 days; 72 months, 336.14 weeks ±10.14 weeks. Infant WLz from 0 to 12 months and BMIz at 72 months were calculated based on the 2000 Centers for Disease Control and Prevention (CDC) infant and child growth charts [39].

#### Covariates

We included several covariates in our model. Maternal socio-demographic characteristics included maternal age, race, marital status, annual household income, and education. Maternal self-reported race was categorized as white vs. non-white. Marital status was dichotomized as married vs. single (which included never married, divorced, separated or widowed). Since the median household income of our sample was $40,000, this variable was dichotomized into ≥ $40,000 vs. <$40,000. The median number of years mothers spent in school was 16 years, therefore, maternal education was dichotomized into ≥16 years (college graduate) vs. < 16 years (some college or below). Maternal pre-pregnancy weight status is associated with infant weight gain and growth, [40] and maternal BMI was divided into one of three BMI categories: normal weight = 18.50–24.99, overweight = 25.00–29.99, and obese ≥30. Gestational diabetes has also been associated with overweight status and was included in the model. [29–31] Mothers were categorized as having diabetes if they reported having gestational diabetes, juvenile onset diabetes, or adult onset diabetes during their pregnancy. Maternal smoking has been associated with infant weight status and overweight risk, [41] and was therefore included in the model. Breastfeeding has been associated with both infant weight status in childhood, [42] as well as risk of infection in children. [43] While the American Academy of Pediatrics (AAP) recommends exclusively breastfeeding for 6 months, most mothers-infant pairs do not exclusively breast feed for that duration. [44, 45] The CDC National Immunization Survey reported that only 18.8% of infants born in 2011 exclusively breastfed for six months while 32.9% exclusively breastfed for four months. [46] Therefore, we included exclusive breastfeeding for 4 months as a covariate. Infant sex was also included in the model.

#### Analysis

Statistical analysis was conducted using SAS 9.4 software (Cary, NC). Means and frequencies were used to describe the sample. Simple unadjusted bivariate analyses were conducted using generalized linear models (GLM) or chi-square statistics to determine whether WLz/BMIz and covariates differed between the three antibiotic groups. Using PROC MIXED in SAS and tested for fixed and random effects, we conducted linear growth modeling of 0 (birth), 2-, 4-, 6-, 12- and 72-month assessments with final models including planned adjustments for covariates described above. First we estimated the unconditional growth model with no covariates to examine the relative fit of random intercept models with and without allowance of individual random slopes. Tests of nested models suggested that adding a random effect for linear slope improved model fit (− 2 log likelihood decreased from 10,920 to 10,611). Next, we compared the relative fit of a linear model that also included a term to capture differences in the rate of acceleration in weight changes over time with a quadratic effect (time^2^). The model with the quadratic effect improved fit significantly (− 2 log likelihood decreased from 10,288 to 10,175) over a model with only a linear effect of time. Therefore, subsequent models included both linear and quadratic effects to describe changes in WLz/BMIz over time. A dummy coded index for antibiotic use group was tested to examine whether there were differences in overall levels of WLz/BMIz across time points (Model 1) and whether interactions between antibiotic group and time suggested that the linear or quadratic rate of change in WLz/BMIz differed across groups (Model 2). The final model (Model 3) included adjustments for covariates known to impact infant/child weight status, including maternal age, maternal BMI, race, marital status, income, education, smoking, diabetes, exclusive breastfeeding for 4 months, and infant sex. Statistical significance was set at *p* ≤ 0.05 for all analyses.

## Results

Children had a mean birth weight of 3.50 kg (S.D. 0.46) (Table 1). By the age of 72 months, 11.09% were either overweight or obese. Antibiotic use ranged from 0 to 8 courses over the first 12 months. The majority of infants (60.58%) did not receive any antibiotics; 33.79% received 1–2 antibiotic courses and 5.63% received 3 or more antibiotic courses during the first year. In total, a little over half the mothers were either overweight (27.82%) or obese (23.55%) at the pre-pregnancy time point. Over half the sample (55.46%) had a college degree or higher, and 30.21% exclusively breast fed for at least 4 months. Demographic characteristics of those who received 0, 1–2, or 3+ antibiotics courses did not differ significantly from each other except for maternal education. Those who received 3+ antibiotic courses were more likely to have a college degree or higher (Table 1).

**Table 1:** Sample Characteristics of children and mothers stratified by antibiotic dose categories (*n* = 586)

	Total Sample n = 586	No antibiotics *n* = 355	1–2 antibiotics *n* = 198	3+ antibiotics *n* = 33	*p*-value
Mother:					
Age (years; mean ± SD)	30.95 (±5.12)	30.95 (±5.07)	31.08 (±5.22)	30.24 (±5.08)	0.69
Gestational weight gain (kg; mean ± SD)	13.58 (±5.96)	13.49 (±5.70)	13.81 (±6.05)	13.10 (±8.02)	0.75
Pre-pregnancy WeightStatus^a^: (%)					
Normal weight	48.63	49.01	48.99	42.42	0.73
Overweight	27.82	27.61	28.28	27.27	
Obese	23.55	23.38	22.73	30.30	
White vs. Other (%)	91.64	90.99	91.41	100.00	0.20
Married vs. Single (%)	88.57	89.01	86.87	93.94	0.46
Income (≥$40,000) (%)	68.77	69.58	65.66	78.79	0.28
Education (≥ college grad) (%)	55.46	57.75	48.99	69.70	0.03
Smoked during pregnancy (%)	5.12	3.94	6.57	9.09	0.23
Gestational Diabetes Mellitus^b^ (%)	9.04	9.30	9.09	6.06	0.82
Exclusive Breast Feeding × 4 mos (%)	30.21	32.85	25.51	30.30	0.20
Vaginal Delivery (%)	69.97	70.42	69.70	66.67	0.90
Infant:					
Sex: Male (%)	48.89	49.15	50.00	39.39	0.52
OW/OB^c^ at 72 months (%)	11.09	8.17	14.65	21.21	0.01
Antibiotic Course: (%)					
0	60.58				
1–2	33.79				
3+	5.63				
Birth weight (kg; mean ± SD)	3.50 (±0.46)	3.49 (±0.45)	3.51 (±0.48)	3.48 (±0.44)	0.78
WL z-score (mean ± SD):					
0 mos	− 0.46 (±1.34)	− 0.43 (±1.35)	− 0.54 (±1.32)	− 0.27 (±1.31)	0.48
2 mos	0.02 (±1.52)	− 0.07 (±1.43)	0.15 (±1.68)	0.38 (±1.37)	0.15
4 mos	0.02 (±1.50)	− 0.08(±1.47)	0.12 (±1.54)	0.48 (±1.53)	0.10
6 mos	0.17 (±1.64)	− 0.02 (±1.59)	0.43 (±1.68)	0.79 (±1.59)	< 0.01
12 mos	0.11 (±1.58)	− 0.01 (±1.57)	0.32 (±1.59)	0.27 (±1.65)	0.11
BMI z-score (mean ± SD): 72 mos	0.31 (±1.23)	0.24 (±1.15)	0.37 (±1.30)	0.67 (±1.55)	0.14

^a^ Maternal pre-pregnancy weight status was classified by the following definitions: normal weight = 18.5–24.99, overweight = 25–29.99, obese ≥30 (mean = 36.03, S.D. 5.40)

^b^ Maternal diabetes was defined as self-report of gestational diabetes, juvenile onset diabetes, or adult onset diabetes during or prior to pregnancy

WL z-score = Weight-for-length z-score

BMI z-score = Body Mass Index z-score

^c^OW/OB = Overweight/Obese; Children were identified as being OW/OB if they had a BMI z-score ≥ 1.64

**Fig. 2:**
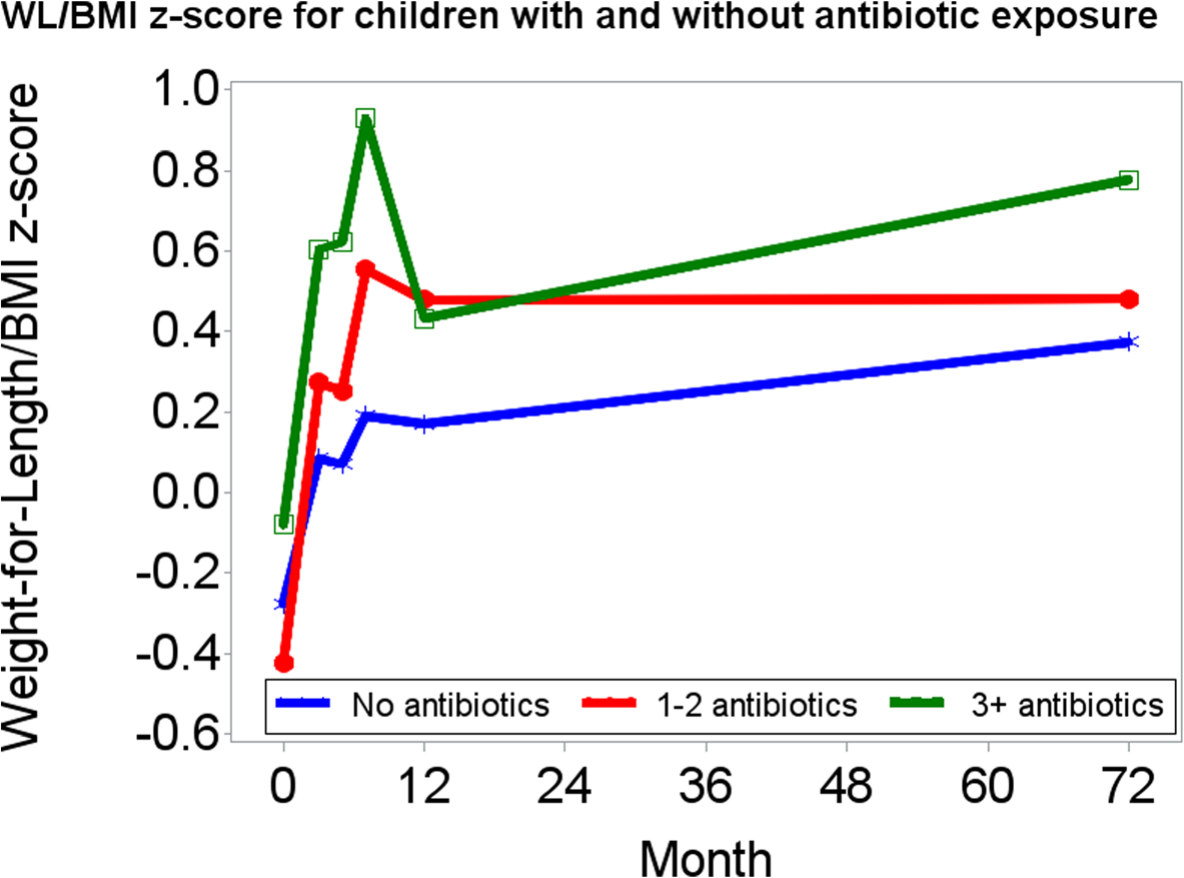
Growth model examining the effect of antibiotic course on Weight-for-Length/BMI z-score from birth to 6 years. Displays the growth trajectories of children from birth to 6 years based on the number of antibiotic courses the child received from birth to 12 months. The model controlled for known covariates for infant/child weight status, including maternal age, maternal BMI, race, marital status, income, education, smoking, diabetes, exclusive breastfeeding for 4 months, and infant sex

In the mixed effects linear growth model, we assessed the effect of linear time, quadratic time and antibiotic course on child growth (Table 2). In the unconditional model (not shown), the Intra Class Correlation coefficient indicated that 25.7% of the variance in growth was accounted for by differences between children. In model 1, we observed significant increases in WLz/BMIz over time (linear time; *p* < 0.0001) and that rates of change in WLz/BMIz differed across 2-, 4-, 6-, 12- and 72-month assessments (quadratic time; *p* < 0.0001). Across assessments, infants who had 3+ antibiotics in the first year had a 0.42 (S.E. 0.16) higher WLz/BMI z-score than infants who had no antibiotics (*p* = 0.009). However in Model 2, we observed a significant interaction between antibiotic group and the quadratic effect of time (*p* = 0.003). Infants who had 3+ antibiotic courses had a more rapid increase in their rate of growth over time (i.e. the quadratic effect; *p* = 0.003). This interaction remained statistically significant after adjusting for covariates in Model 3. Figure 2 presents covariate-adjusted changes in WLz/BMIz over 2-, 4-, 6-, 12-, and 72-month assessments for infants in the antibiotic exposure groups.

**Table 2:** Linear growth model of Weight-for-Length/BMI z-score from birth to 72 months based on antibiotic exposure during the first year of life (*n* = 586)

	Model 1		Model 2		Model 3	
	Estimate (S.E)	*P*-value	Estimate (S.E)	P-value	Estimate (S.E)	P-value
Initial WL/BMI z-score (intercept)	− 0.21 (0.05)	< 0.0001	− 0.18 (0.05)	0.0006	−0.10 (0.35)	0.77
Linear time (months)	0.06 (0.007)	< 0.0001	0.04 (0.009)	< 0.0001	0.04 (0.009)	< 0.0001
Quadratic time (months2)	−0.0007 (0.0001)	< 0.0001	− 0.0005 (0.0001)	< 0.0001	− 0.0004 (0.0001)	< 0.0001
Antibiotic course		0.009		0.09		0.10
0	0		0		0	
1–2	0.17 (0.08)		0.10 (0.09)		0.08 (0.09)	
3+	0.42 (0.16)		0.37 (0.18)		0.38 (0.18)	
Linear Time x Antibiotic course				0.003		0.002
0			0		0	
1–2			0.05 (0.02)		0.05 (0.02)	
3+			0.04 (0.03)		0.04 (0.03)	
Quadratic Time x Antibiotic course				0.003		0.002
0			0		0	
1–2			−0.0007 (0.0002)		−0.0008 (0.0002)	
3+			−0.0005 (0.0004)		−0.0005 (0.0004)	

The models presented here included a random statement for the individual allowing variation in intercept, time, time2, and antibiotic course with a variance components (VC) covariance structure between the effects using a Maximum Likelihood Estimation method. Model 1 includes the effect of time, time2, and antibiotic dose on weight-for-length/BMI z-score changes from birth to 72 months. In Model 2, we included the variables for time x antibiotic course and time2x antibiotic course. Model 3 controlled for known covariates including maternal age, maternal BMI, race, marital status, income, education, smoking, diabetes, exclusive breastfeeding for 4 months, and infant sex. Entries show parameter estimates and standard errors in parentheses.

## Discussion

In this observational cohort study, we found that antibiotic use was independently associated with increased growth during early childhood. The dose-response relationship between antibiotic exposure and child weight status, demonstrated in our study and supported by prior studies [5, 6, 13, 24, 26] provides additional support for judicious antibiotic prescribing during early childhood. Finally, evidence of a relationship between antibiotic exposure and growth trajectory in a US cohort with good breastfeeding ascertainment is a key contribution given that breastfeeding has not been included as a covariate in prior US studies. [5, 6, 12, 24]

It is important to consider that this association held true while controlling for exclusive breastfeeding status, a factor thought to affect weight gain in infancy, antibiotic prescribing, and gut microbiota composition, and the impact of antibiotics on the gut microbiome. [27, 47] Breastfeeding status can be challenging to determine from health records and therefore, was included in some [7–9, 25] but not all prior studies in this area. [5, 6, 13, 24] Since the postulated mechanism leading to the association between antibiotic exposure and weight status is through the gut microbiota, and breastfeeding is an important early contributor to the gut microbiota, [48] inclusion of breastfeeding is key to understanding this relationship. A study in Finland found that antibiotic exposure while breastfeeding may attenuate the beneficial effects of exclusive breastfeeding, [34] i.e., children who are exposed to antibiotics may not have the lower weight that would be anticipated of breastfed babies.

The dose response effect that we found between antibiotic exposure and weight status supports the findings of the majority of the cohorts that evaluated this relationship previously. A study in Finland examining a population-based cohort of children under 24 months old found that children had a 0.18 higher BMIz when exposed to ≥4 antibiotic courses between 0 and 24 months compared to a 0.10 higher BMIz in those exposed to 2–3 courses. [13] In Central Pennsylvania, there was a similar dose-response effect for antibiotic exposure and child BMI when the child’s antibiotic exposure was ≥4 doses for children in the first three years of life. [24] Additionally, a study conducted among older children (3–18 years old) in the same Pennsylvania health system found a dose-response relationship with the BMI trajectory of those who received > 7 antibiotic courses compared to those who received fewer courses. [6] Another study among children < 5 years old in urban health centers in Philadelphia found that children also did not exhibit a dose response relationship until they received ≥4 courses of antibiotics in the first 24 months of life. [5] Interestingly, a study in Canada did not find a dose response relationship between antibiotic exposure at < 12 months and overweight status by the time children were 12 years old. [7] This result may reflect differences in how the exposure was assessed (i.e., medical record data vs. provincial prescription records).

For an average height 72 month old girl in our cohort (114 cm), a child exposed to ≥3 courses of antibiotics in the first year of life would have a 0.82 kg higher weight than a child who was not exposed to antibiotics in the first year. This would lead to a 0.4 BMIz score difference between the two children. While this difference may be subtle at 72 months old, childhood overweight at 60 months old tracks into adolescence [49] and adulthood [50], therefore, a higher BMIz score at 72 months places children at a higher risk of overweight in adulthood. We chose to model growth trajectories over time because it allowed for a more sensitive analysis of the observed differences at 72 months while controlling for the correlation over time between each subject’s weight and height data. Further studies are warranted to follow children into adulthood to determine the life course implications of early and continued childhood antibiotic exposure.

While this study adds to the growing literature that antibiotic use in infancy may affect weight status in childhood, there were limitations. The study includes 586 children, and the demographics of the sample are not representative of the broader US population. [35] This study was a retrospective observational study that depended on parental recall of the child’s height and weight from the doctor’s office, and antibiotic receipt in the last 2 weeks. [35] Recall bias could impact the exposure and outcome in our study. However, the proportion of children with no antibiotic exposure in the first year of life is similar in this cohort as has been reported in other studies, [51, 52] and the proportion of children who were overweight/obese is lower than is reported nationally. Further, each of the scales utilized, and the individuals completing the measurements were at different clinical sites which likely contributed to variability in anthropometric data. This study does include data on the frequency of antibiotic use during the first 12 months, which allowed us to examine the dose-response relationship. However, we may be underestimating the effect of antibiotics because mothers were not asked to record any interceding antibiotic courses the child may have received prior to the past 2 weeks. Furthermore, they were not asked to report on antibiotic use between 12 months and 6 years, limiting our ability to examine the effect of continued antibiotic use on later weight status or weight gain trajectories. Unfortunately, we were also unable to report on the type of antibiotic the child received, or the duration of antibiotic use. This information may have been useful since prior research reports that broad spectrum antibiotics are associated with a higher risk of obesity development. [5] Given these limitations, a prospective study is warranted to more clearly define the relationship between antibiotic use and weight status.

## Conclusions

Children exposed to antibiotics prior to one year of age demonstrated a dose-response effect on growth trajectories while adjusting for key covariates. This finding contributes to the growing body of literature that suggests that antibiotic exposure may be contributing to the prevalence of obesity among children, and demonstrates a key area for physicians to intervene. While we generally think of early childhood as an important time to treat infections due to the vulnerability of children with an underdeveloped immune system, the cost of treatment may incorporate risk for other conditions including obesity and obesity-associated chronic disease. Therefore, judicious use of antibiotics, especially during the first year of life, and acknowledging the risk of obesity with administration is warranted and required conversation between physicians and parents.

## Abbreviations

(IBD): Inflammatory bowel disease
6YFU: 6 year follow-up study
AAP: American Academy of Pediatrics
BMIz: BMI z-score
CDC: Centers for Disease Control and Prevention
IFPS II: Infant Feeding Practices II
WLz: Weight/Length z-score
